# Extracellular matrix shapes cancer stem cell behavior in breast cancer: a mini review

**DOI:** 10.3389/fimmu.2024.1503021

**Published:** 2025-01-09

**Authors:** Lei Li, Yidan Tang, Ling Qiu, Zhengrui Li, Ruo Wang

**Affiliations:** ^1^ Department of Laboratory Medicine, Peking Union Medical College Hospital, Peking Union Medical College & Chinese Academy of Medical Science, Beijing, China; ^2^ State Key Laboratory of Complex Severe and Rare Diseases, Peking Union Medical College Hospital, Peking Union Medical College & Chinese Academy of Medical Science, Beijing, China; ^3^ Faculty of Medicine, University of Debrecen, Debrecen, Hungary; ^4^ Shanghai Jiao Tong University School of Medicine, Shanghai Jiao Tong University, Shanghai, China; ^5^ Shengli Clinical Medical College of Fujian Medical University, Department of Breast Surgery, Fujian Provincial Hospital, Fuzhou University Affiliated Provincial Hospital, Fuzhou, China

**Keywords:** extracellular matrix, cancer stem cells, breast cancer, ECM, BCSCs

## Abstract

Today, cancer has become one of the leading global tragedies. It occurs when a small number of cells in the body mutate, causing some of them to evade the body’s immune system and proliferate uncontrollably. Even more irritating is the fact that patients with cancers frequently relapse after conventional chemotherapy and radiotherapy, leading to additional suffering. Scientists thereby presume that cancer stem cells (CSCs) are the underlying cause of metastasis and recurrence. In recent years, it was shown that not only can chemotherapy and radiotherapy underperform in the treatment of breast cancer, but they can also increase the number of breast cancer stem cells (BCSCs) that transform regular breast cancer cells into their own population. Such data somewhat support the aforementioned hypothesis. Meanwhile, our understanding of the extracellular matrix (ECM) has changed considerably over the last decade. A lot of studies have bit by bit complemented human knowledge regarding how the ECM greatly shapes the behaviors of BCSCs. In this review, we highlighted the influence on BCSCs exerted by different critical components and biochemical properties of ECM.

## 1 Introduction

Cancer is a significant issue affecting public health on a global scale, and the majority of malignancies diagnosed in humans are currently incurable illnesses. According to the most recent data available from the National Cancer Institute (1), breast cancer is the leading cause of morbidity among women with cancer. Even though there has been a significant advancement in the early diagnosis of breast cancer and increasing availability of treatments for breast cancer, such as surgical resection, radiotherapy, endocrine therapy, immunotherapy (2–5)., the prognosis for patients continues to be poor due to the high incidence of distant metastases from breast cancer.

The notion of cancer stem cells (CSCs) is garnering an increasing amount of interest as research into the disease continues to make strides forward. Cancer stem cells are cancer cells that have stem cell properties, that is, they have the ability to differentiate and self-renew. Tumor stem cells are tumorigenic-seeded cells that are more resistant to radiation and endocrine therapy than conventional tumor cells. They are also the primary source of metastasis development, which occurs when a tumor spreads to other parts of the body (6). It is also a significant component that results in recurrence following clinical therapy and has an impact on the percentage of patients who can live disease-free as well as their overall survival rates. Recent research has shown that targeting CSCs may be useful for the treatment of malignancies, including breast cancer (7). This indicates that a deeper knowledge of the characteristics of CSCs may be necessary for the eradication of CSCs. However, further research is needed to figure out how breast cancer stem cells (also known as BCSCs) manage to keep their basic characteristics.

The tumor microenvironment where malignant tissue grows is the external environment for the growth of tumor cells and tumor-associated cells. As an important component of the tumor microenvironment, the dysregulation of various components and biological properties of the tumor extracellular matrix (ECM) can not only directly regulate the fate of such stem cells, but also the interaction between them may establish the abnormal feedback loops, eventually better the cancer cells proliferation (8, 9).

**Figure 1:**
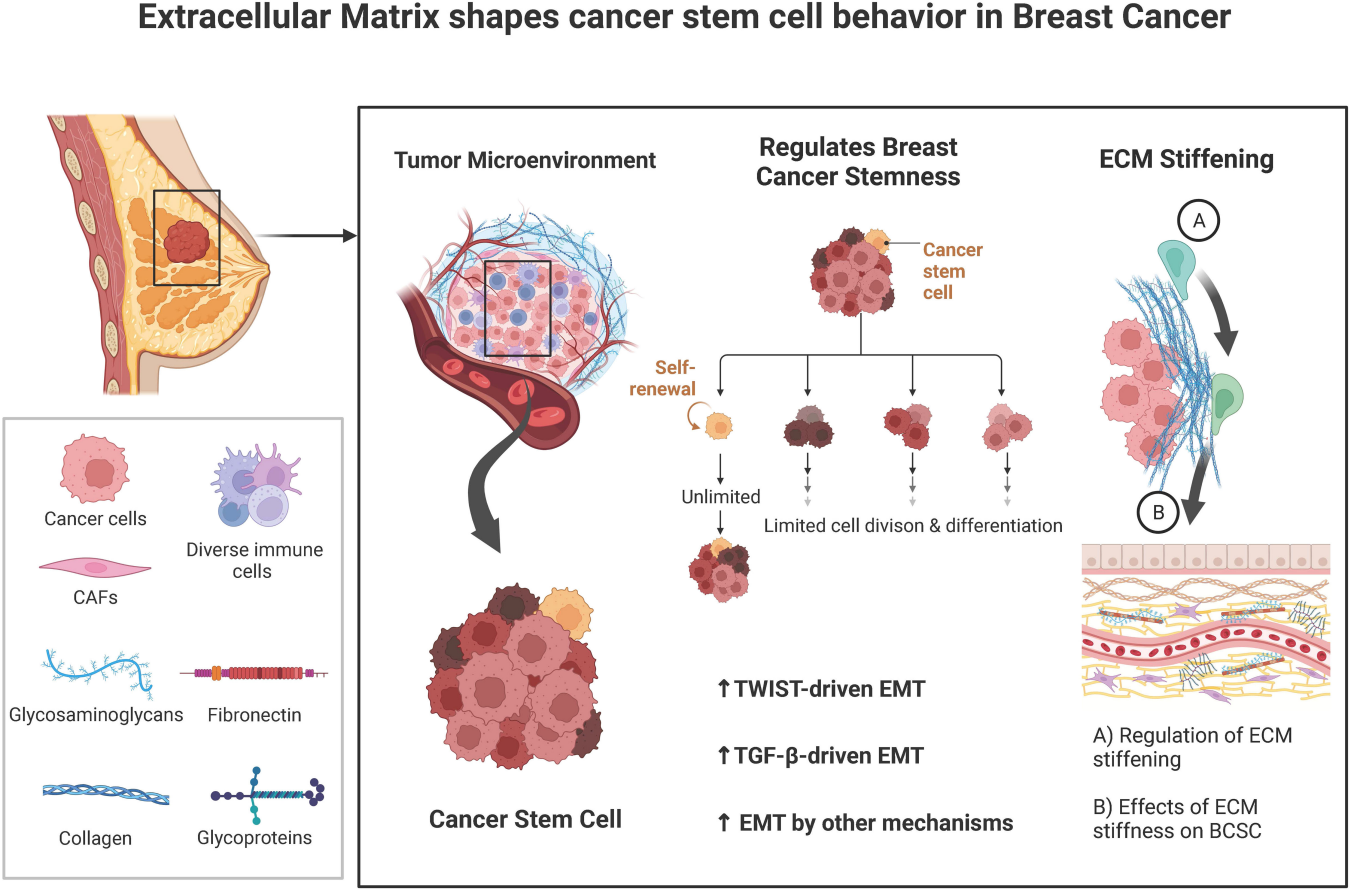
A brief summary of the known interplays between tumor extracellular matrix (ECM) and cancer stem cells (CSCs).

In this review, we investigated the interactions between ECM and CSCs, which might give many useful hints to address this extremely malignant subgroup of cancer cells in breast cancer and proposed certain novel insights into the development of breast cancer treatment (**Figure 1**).

## 2 The influence of the components of ECM on BCSC

The ECM is a relatively dynamic and intricate three-dimensional reticular molecular structure surrounding cells within tissues, composed of an interstitial matrix and basement membrane (BM) (10). The basement membrane surrounds the mammary gland and is the main ECM that mediates the remodeling of the tumor microenvironment and induces BCSCs formation and maintenance. Hynes and colleagues analyzed the human and mouse genomes using different proteomics techniques and reported the most comprehensive list of proteins defining the mammalian ECM to date. About 300 of these proteins constitute its core matrix, consisting of 43 collagen subunits (Collagen), 36 proteoglycans (PGs), and about 200 complex glycoproteins (including laminin, elastin). In addition, many ECM-related proteins are not part of the ECM but have important roles in ECM remodeling, including growth factors, cytokines, mucins, secreted C-type lectins, galactogens, plexins, and ECM-modifying enzymes (e.g., transglutaminase, lysyl oxidase, and hydroxylase) (11).

### 2.1 Collagen

Collagen is the main structural protein of the ECM and is divided into fibrillar (collagen I-III, V, and XI) and non-fibrillar types. Malignant breast cancer is associated not only with a significant increase in collagen content, but also with a substantial change in its composition, including increased fibrillar collagens I, III, and V accumulation, among them, type I collagen (Col-I) is its most abundant component, which may be related to tumor migration and aggressive characteristics. Although Iuri C. Valadão’s study showed that Col-I enrichment failed to induce the proliferation of BCSCs cells and the conversion to mammospheres *in vitro* (12), it was essential for maintaining BCSC-related functional activity characteristics, such as increased expression of the pluripotency factors Nanog and Oct-4 was founded in a Col-I-enriched culture environment, which coordinately determines BCSC self-renewal (13). In addition, high type I collagen density can modulate metabolic shifts in breast cancer cells, as evidenced by reduced glucose oxidation and increased glutamine utilization in the TCA cycle, while increased glucose flux may be shifted to the serine synthesis pathway, and this metabolic plasticity is a key aspect of cellular metastatic potential in BCSCs (14, 15).

Overexpression of BCSCs-related properties was found in the breast cancer cell line MCF-7 cells cultured in a collagen-rich *in vitro* three-dimensional (3D) cell culture model compared to the conventional two-dimensional (2D) cell culture system, possibly due to the formation of a hypoxic microenvironment induced by the collagen scaffold, which in turn upregulated the expression of hypoxia-inducible factors (HIFs) and EMT-related transcription factors ultimately leading to cancer cell dedifferentiation (16). Collagen can also direct the directional migration of BCSCs by directly contacting them. For example, the ability of BCSCs in breast cancer to move directionally on well-aligned collagen is significantly enhanced, which may be due to the smaller size of BCSCs are smaller than other tumor cells and have higher phenotypic plasticity and outward protruding motility (16). Apart from that collagen can also enhance the metastatic potential of BCSCs by activating some tumor-associated signaling pathways, such as PI3K/AKT/mTOR, RTK/RAS/MAPK. The rigid ECM formed by high-density collagen enhances the stemness of ERα+ breast cancer CSC and promotes the formation of lung metastatic foci by activating the AKT-mTOR signaling pathway (17).

The increased collagen density at the tumor-stroma interface directs the establishment of a stem-like phenotype through secreted diffusion factors (Hedgehog paracrine signaling), which may confirm that triple-negative breast cancers with worse prognosis exhibit simultaneous activation of Hedgehog pathway components and fibrillar collagen program compared to patients with other breast cancer subtypes (18).

### 2.2 Glycosaminoglycans

Glycosaminoglycans (GAGs, also called mucopolysaccharides) are polymeric sugar chains formed by a large number of monosaccharides interconnected by glycosidic bonds. The GAGs most closely related to CSC in ECM is Hyaluronic Acid (HA) since the HA is found at high levels in the stem cell niche (19). The increase in the size of breast cancer tumor spheroids after HA hydrogel treatment symbolizes its important role in maintaining the stemness of CSCs. Theerawut et al. demonstrate that HA allows breast cancer cells to return to a stem cell state via EMT by modulating the TGF-β network (20). Recent studies have shown that using different molecular weights of HA and controlling the duration of HA treatment of cells may be associated with different aggressive characteristics of BCSCs. After treating breast cancer cells with high molecular weight (HMW) and low molecular weight (LMW) HA, respectively, only LMW-HA was found to increase the secretion of IL-8, which has been shown to induce the expression of EMT and stem cell genes at high levels. Also, LMW-HA could activate the Wnt pathway by inducing the nuclear internalization of β-linked protein, which ultimately promotes mesenchymal and stem cell phenotypes. In addition, HA hydrogel treatment of breast cancer cells for 3 days revealed increased expression of EMT markers. Whereas the prolonged presence of HA in the tumor microenvironment was associated with a decrease in EMT expression, this is not controversial, as activation of EMT genes was previously reported to be transient during tumor invasion and metastasis.

CD44, a cell surface receptor for HA has also been reported as an important CSC marker. BCSCs cells enriched with HA hydrogels highly express CD44 molecules and exhibit enhanced tumorigenicity, metastatic potential, and chemoresistance (21). Earlier studies have shown that HA interaction with CD44 mediates increased nuclear expression and strong co-localization of STAT3 and Nanog, which subsequently affects the expression of downstream target genes (e.g., pluripotent stem cell regulators. Rex1 and Sox2, multidrug resistance gene 1), and aberrant expression of these genes is possibly associated with the tumorigenic potential of stem cells (22) and multi-drug resistance, it can be suggested that HA-CD44 interactions may act as upstream activator signals that mediate the specific behavior of tumor cells being reprogrammed with tumor stem cells (23). In addition, the resistance of BCSCs to anticancer therapy may also be achieved through the activation of the CD44-NRF2 axis.

In addition, BCSCs phenotype and resistance to anticancer therapy may also be achieved through activation of the CD44- Nuclear factor erythroid 2-like 2 (NRF2) axes. Previous studies have shown that antioxidant and detoxification proteins are significantly abundant in NRF2^high^CSCs from breast tumors after proteomic analysis (24). As supportive evidence, a study by Tongde et al. revealed that the NRF2 knockdown in mammospheres was responsible for the attenuation of tumorigenicity and chemoresistance (25).

It should be noted that the potential contribution of CD44 to the maintenance of higher levels of ROS metabolism in CSCs than in normal cells is of increasing interest, as lower levels of ROS are found in subpopulations of CSCs in human and murine breast tumors compared to the corresponding non-tumorigenic cells (26). It should be noted that the potential contribution of CD44 to the maintenance of higher levels of ROS metabolism in CSCs than in normal cells is of increasing interest, as lower levels of ROS are found in subpopulations of CSCs in human and murine mammary tumors compared to the corresponding non-tumorigenic cells, and hyaluronan/CD44s-mediated activation of NRF2 may be a programming mechanism for enhanced ROS defense in CSCs (27).

Several previous studies have shown that HA also promotes metabolic shifts associated with CSC-like features in breast cancer cells by interacting with a variety of signaling molecules. Excessive HA production has been reported to increase HIF-1, a potential regulator of glycolysis in breast cancer cells, thereby accelerating the flux of the hexosamine biosynthesis pathway (HBP) and glycolysis, and this metabolic shift to aerobic glycolysis reflects the specific characteristics of the undifferentiated and self-renewing state of cancer cells and inhibition of the HA-HIF-1 axis similarly reduces the CSC-like subpopulation in breast cancer cells (28).

Another study by Heena et al. points out that HA-induced upregulation of the cystine-glutamate exchange protein transporter protein xCT in CSC isolated from MDAMB-231 cells, subsequently causing a significant reduction in intracellular glutamate secretion, and promoting its intracellular anaplerosis to bypass the oxidative metabolism of sugars and lipids for energy (29).

### 2.3 Glycoproteins

Glycoproteins are stable compounds formed by the covalent binding of proteins to sugars, whose main role is to interconnect the components of the ECM and the ECM with neighboring cells to regulate downstream signaling. Among them, those closely related to CSC are adhesion glycoproteins such as laminin and fibronectin.

#### 2.3.1 Laminin

Previous evidence from the literature validates laminin-promoted tumorigenic properties of sorted cancer stem cells population from breast cancer. Laminin provides a looser environment for breast stem cell attachment than collagen or other matrix proteins, and it is because of its lower adhesion properties that stem cells in a laminin-rich environment maintain their self-renewal properties (30).

It has also been shown that the binding of laminin to its specific receptor mediates the activation of Hippo transducer TAZ, which helps to maintain the dryness of breast cancer CSC while promoting the secretion of more laminin by BCSC and thus constitutes a positive feedback loop (31).

However, Damián Eet al. showed that the overexpression of laminin caused a reduction in stem cell compartments in breast cancer cell lines by activating the MAPK/ERK signaling pathway (32). The reason for the controversy among different studies may be related to the differences between the selected experimental models and the culturing environment.

#### 2.3.2 Fibronectin

Fibronectin has multiple functions in organisms, including but not limited to: promoting cell adhesion and proliferation, repairing damaged cells, stimulating cells to secrete multiple functional proteins, regulating the dynamic balance between cells and intercellular matrix, improving microcirculation inside and outside cells, ensuring the transport of internal and external substances, binding to the surface of lymphocytes. Fibronectin overexpression was found to be associated with the metastatic microenvironment of tumors in breast cancer cases and similarly, Sharmistha et al. showed that breast cancer CSC cells cultured in a fibronectin-rich environment exhibited enhanced expression of Zeb1, a transcriptional protein associated with breast cancer metastasis and that the fibronectin scaffold induced EMT-like transformation of breast cancer CSC cells through activation of TGF-β and its downstream target genes (30).

## 3 Effects of the biochemical properties of ECM on BCSCs

The extracellular matrix (ECM) is a sophisticated system of proteins and other substances that surrounds the cells in tissues and provides support for them. Together, such components determine the biochemical properties of ECM in breast cancer. Breast cancer stem cells (BCSCs) meanwhile are a comparatively limited subpopulation of cells within breast tumors that are capable of continuously self-renewing and differentiating into a variety of other cell types. They are nowadays widely presumed to play a significant role in tumorigenesis, metastasis, and resistance to treatment. Numerous studies over the past decades have showcased the potential or well-proven connections between the two.

### 3.1 ECM regulates breast cancer stemness via EMT

Epithelial-mesenchymal transition (commonly defined as EMT) is a process that can transform divided cells back into CSCs. Epithelial-mesenchymal transition is a process in which epithelial cells loss polarity and intercellular connections and acquire mesenchymal cell-like assault and motility capabilities. This process is responsible for increasing the stemness of malignancies (33). The control of the EMT process involves a wide variety of ECM components as well as signaling pathways. Among them, TWIST and TGF- have distinguished themselves as the EMT’s master regulators. The expansion of the CSC population, the spread of the tumor, and resistance to chemotherapy are all caused by ECM that activates TWIST and/or TGF-driven EMT. Upregulation of the expression of TWIST and TGF- is facilitated by COL11A1, EDB-FN, and serglycin, respectively. In addition, HA and stiff matrix can activate EMT along both axes. It is interesting to note that BCSCs go through a partial EMT and display a hybrid E/M state. This state has properties of both mesenchymal and epithelial cells, which helps to boost the adaptability of BCSCs. Epithelial-like BCSCs exhibit a proliferative capacity that favors the colonization of tumors at secondary metastatic sites, in contrast, to mesenchymal-like BCSCs, which promote stemness and have higher metastatic ability. Mesenchymal-like BCSCs also have a higher ability to spread cancer to other parts of the body (34, 35).

#### 3.1.1 ECM that activates TWIST-driven EMT

In breast cancer MCF7 cells, the TWIST is a master transcription factor that promotes the epithelial-mesenchymal transition (EMT) as well as the production of BCSC markers (CD44, ALDH1). In addition, Vesuna et al. demonstrated that TWIST can promote BCSC expansion independently of EMT by interacting with E-box sequences in the CD24 promoter (36). This results in decreased expression of CD24, which is a characteristic of BCSC (CD44+/CD24-/low, ALDH+). TWIST promotes BCSC expansion by interacting with E-box sequences in the CD24 promoter. As a result, ECM that boosts TWIST expression can stimulate the production of BCSC (37). It has been demonstrated that cancer-associated fibroblasts (CAF) create a kind of collagen called collagen type XI alpha1 (COL11A1), which has been proven to upregulate TWIST and may thus contribute to the cancer stemness of the disease. Its expression is extremely low in normal breast stromal cells, but overexpression of COL11A1 in breast cancer is linked to both local invasion and metastasis. In addition, BCSCs have been shown to have high quantities of the enzyme hyaluronan synthase 2 (HAS2) as well as the additional domain B of fibronectin (EDB-FN) (38). The overproduction of hyaluronic acid (HA) by HAS2 causes an increase in the BCSC population through TWIST-driven EMT (39). The function of EDB-FN in the control of EMT and stemness is established by the knockdown of EDB-FN by siRNA in BCSC, which results in a decrease in the expression of TWIST and BCSC markers (20). This demonstrates that EDB-FN is involved in the regulation of EMT and stemness (CD44 and ALDH1). Additionally, TWIST may be triggered by increased matrix stiffness, which enhances the nuclear translocation of TWIST by freeing it from cytoplasmic binding protein G3BP2 and allowing it to go into the nucleus (40). An EPHA2/LYN/TWIST1 signaling pathway was established by Fattet et al., in which a rigid matrix leads to successive phosphorylation of ephrin receptor EPHA2, LYN kinase, and TWIST. After being phosphorylated, TWIST detaches from G3BP2 and travels to the nucleus, where it induces EMT in breast cancer cells (41).

#### 3.1.2 ECM regulates TGF-β-driven EMT

TGF-, which has some similarities to TWIST, plays a crucial part in initiating EMT, which in turn helps to enhance the BCSC population. TGF- may be generated by both stromal cells (such as CAF) and cancer cells in order to upregulate transcription factors that are involved in EMT (such as ZEB1/2 and Snail) (42). However, the expression of TWIST cannot be increased by TGF-. The epithelial-to-mesenchymal transition (EMT) can be facilitated by hyaluronic acid (HA) (43). In triple-negative breast tumors, the intracellular proteoglycan serglycin (SRGN), which has the potential to be released into the extracellular matrix, is seen in high levels (TNBC) (20). The interaction between SRGN and CD44 on BCSC results in the phosphorylation of CREB1 and an increase in the production of TGF-. In addition to this, TGF- also raises the expression of SRGN by phosphorylating Smad2/3, which then activates the gene. As a result, SRGN and TGF- combine to generate a constructive feedback loop that promotes EMT as well as cancer stemness. On the other hand, asporin prevents TGF- from directly binding to Smad2 and activating it, which causes the activation of Smad2 to be halted (44, 45). As a consequence, asporin is a proteoglycan that lessens stemness and is linked to a favorable prognosis. A high amount of IL-1 suppresses asporin synthesis from CAFs, which results in a lower level of asporin expression in TNBC compared to hormone receptor-positive tumors, which have greater levels of asporin expression. In addition, the degree of elasticity of the extracellular matrix (ECM) determines whether the tumor-suppressing or tumor-causing properties of TGF- are expressed (46). Apoptosis can be induced by TGF- in the soft matrix of normal breast tissue as well as breast tissue that is in the early stages of malignancy. In contrast, when cancer advances and the matrix becomes more rigid, TGF- changes its function to accelerate EMT. This occurs simultaneously with the progression of the disease (47).

#### 3.1.3 ECM regulates EMT by other mechanisms

EMT can be regulated by a number of other pathways in addition to those controlled by TWIST or TGF-, which are the two most well-known examples. Restin is an energetic fragment that was produced from collagen XV. Researchers Lu and colleagues have proven that restin can prevent EMT in breast cancer by binding to p73, activating miR-200a/b, and thereby decreasing the production of ZEB1/2, which are master transcription factors for EMT (48). Glypican-3 (GPC3) is a proteoglycan that has a high level of expression in normal breast tissue but a lower level of expression in breast cancer (49). In a manner analogous to that of restin, GPC3 is capable of downregulating ZEB1 (50). This, in turn, is responsible for reversing EMT, which is characterized by decreased expression of mesenchymal markers (vimentin, N-cadherin), and increased expression of E-cadherin. Consequently, as GPC3 can induce mesenchymal-to-epithelial transition (MET), it can lower the invasive and metastatic ability of breast cancer cells. Exosomes are lipid vesicles that are formed from a wide variety of cell types and are included as one of the components of the extracellular matrix (ECM) (e.g., CAF) (51). Exosomes can transport both microRNA (miRNA) and proteins between cells, which helps to enhance communication between the cells. Exosomal miRNAs (miR-21, miR-378e, and miR-143), transferred from CAF to breast cancer cells, have been found to promote EMT and stemness, which are characterized by higher levels of EMT markers (snail and zeb) and BCSC markers (oct3/4, nanog, and sox2) (52).

### 3.2 ECM stiffness

The stiffness of the extracellular matrix (ECM), which is governed by the content and crosslinking of ECM proteins like collagen and fibronectin may significantly influence the behaviors of BCSCs (47). According to the findings of Paszek MJ et al. in 2005, BCSCs cultured on stiff ECM substrates tend to proliferate more quickly and have greater levels of stemness-related gene expression than breast CSCs cultured on soft substrates. They also discovered certain ECM proteins, such as collagen and fibronectin, that contribute to the rigidity of the ECM (53). Interestingly, this process is activated via ERK activation/ROCK increase.

#### 3.2.1 Regulation of ECM stiffness

The interaction between cancer-associated fibroblasts (CAFs) and cancer cells, which may be broken down into three categories of processes, is responsible for the stiffening of cancer. (1) Both CAFs and cancer cells are responsible for the production of matrix proteins (e.g., collagen, fibronectin). (2) The contraction of the matrix brought on by CAFs contributes to an even greater increase in matrix stiffness. (3) Matrix crosslinking is caused by lysyl oxidase (LOX), an enzyme that is generated by CAFs in addition to cancer cells. Gilkes et al. have demonstrated that breast cancer cells exposed to hypoxia possess an increased level of extracellular matrix (ECM) stiffness (47). This occurs as a result of HIF-1 activating the expression of collagen hydroxylases known as P4HA1, P4HA2, and PLOD2. These enzymes are necessary for the deposition and alignment of collagen. This alignment of the collagen encourages the migration of breast cancer cells, which in turn encourages the invasion and spread of the disease (54).

#### 3.2.2 Effects of ECM stiffness on BCSC

In addition to increasing stemness through the TWIST and TGF—driven EMT pathways, the stiff matrix also activates the ILK/PI3K/Akt signaling pathway. This results in an increase in the expression of BCSC markers (CD44, ALDH, Nanog, and CD49f), which in turn promotes BCSC survival and proliferation. Integrin-linked kinase (ILK), which is a cytoplasmic protein that interacts with 1-integrin, is a critical molecule in the formation of BCSC that is caused by a stiff matrix. This was demonstrated by the fact that RNA interference of ILK was unable to promote the development of BCSC on a stiff matrix. The enhanced sensitivity of breast cancer cells to the stiff matrix is likely the reason for the additional enhancement of the ILK signaling pathway that occurs due to hypoxia in the stiff matrix (55).

## 4 Conclusions and perspectives

Today, cancer has become one of the most severe catastrophes in healthcare while for most cases, effective treatment strategies are still in-short. Cancer as a malignancy is thought to be originated from a small group of cells in the body that accumulatively mutate, which keep on transforming phenotypically, and finally enabling themselves to avoid regular removal by the immune system. One even more frustrating fact is that such malignancies tend to recur after traditional chemotherapy and radiotherapy, usually resulting in severe damage to the human body. On the other hand, a lot of solid tumors (56, 57), breast cancer in particular, conventionally is believed to be organized in a hierarchical way, with cancer cells as the major cell population and cancer stem cells (CSCs) as a minority, while such CSCs seem to be the only reason that accounts for the breast cancer relapses. From this end, some advanced studies reported that manipulating cancer stem cells (CSCs) might serve as a promising strategy in the treatment of certain cancer types, including breast cancer (7). This leads to a belief that more in-depth studies of CSC characteristics are necessitated for its eradication. Interestingly, Extracellular matrix (ECM) as a rising star in precision medicine for various tumors, has raised much attention. The potential link between the aforementioned is of great interest to both the general public and the scientific community. In fact, there are still many difficulties and gaps in the study of ECM and BCSCs. First, due to the extreme complexity and high variability of ECM components, it is difficult to explore the relatively constant interaction between ECM and BCSCs through the combination of experiments and omics. Second, a large number of studies have focused on the promotion of ECM on BCSCs, while relatively ignoring the possible inhibitory effect of ECM on BCSCs, which may be very important. Third, there are still few specific treatments for ECM and BCSCs. In reality, research on ECM and CSC should not be limited to mechanism exploration. In terms of treatment, interfering with ECM to interfere with CSC and thus exert a therapeutic effect may be a feasible solution. However, future research still needs to focus on: 1. Are there sufficiently good specific ECM-related targets? If so, what is the direction of drug development (small molecules, antibodies or others)? 2. For different molecular typing, how to determine whether effective and accurate treatment measures can be provided?

In a nutshell, through this mini review, we inspected the possible ways in which ECM may shape breast cancer stem cells (BCSCs), namely by its essential components like collagen, glycosaminoglycans (GAGs), and glycoproteins, and distinct biochemical properties such as stiffness, aberrant signaling pathways, and more, sharing new insights for future optimization in therapeutic approaches.
